# Long-Term Corticosterone Exposure Decreases Insulin Sensitivity and Induces Depressive-Like Behaviour in the C57BL/6NCrl Mouse

**DOI:** 10.1371/journal.pone.0106960

**Published:** 2014-10-13

**Authors:** Eva L. van Donkelaar, Koen R. D. Vaessen, Jodi L. Pawluski, Annerieke S. Sierksma, Arjan Blokland, Ramón Cañete, Harry W. M. Steinbusch

**Affiliations:** 1 School for Mental Health and Neuroscience, Faculty of Health, Medicine and Life Sciences, Maastricht University, Maastricht, The Netherlands; 2 Central Laboratory Animal Research Facility, Faculty of Veterinary Medicine, University of Utrecht, Utrecht, The Netherlands; 3 University of Liege, GIGA-Neurosciences, Liège, Belgium,; 4 Department of Biological Sciences, Irvine Hall, Ohio University, Athens, Ohio, United States of America; 5 VIB Center for the Biology of Disease, Leuven, Belgium; 6 Center for Human Genetics, KU Leuven, Leuven, Belgium; 7 Department of Neuropsychology and Psychopharmacology, Faculty of Psychology and Neuroscience, Maastricht University, Maastricht, The Netherlands; 8 Unidad de Endocrinología Pediátrica del Hospital Universitario Reina Sofía, Instituto Maimónides de Investigación Biomédica de Córdoba (IMIBIC), Córdoba, Spain; University of Lancaster, United Kingdom

## Abstract

Chronic stress or long-term administration of glucocorticoids disrupts the hypothalamus-pituitary-adrenal system leading to continuous high levels of glucocorticoids and insulin resistance (IR). This pre-diabetic state can eventually develop into type 2 diabetes mellitus and has been associated with a higher risk to develop depressive disorders. The mechanisms underlying the link between chronic stress, IR and depression remains unclear. The present study aimed to establish a stress-depression model in mice to further study the effects of stress-induced changes upon insulin sensitivity and behavioural consequences. A pilot study was conducted to establish the optimal administration route and a pragmatic measurement of IR. Subsequently, 6-month-old C57BL/6NCrl mice were exposed to long-term oral corticosterone treatment via the drinking water. To evaluate insulin sensitivity changes, blood glucose and plasma insulin levels were measured at different time-points throughout treatment and mice were behaviourally assessed in the elevated zero maze (EZM), forced swimming test (FST) and open field test to reveal behavioural changes. Long-term corticosterone treatment increased body weight and decreased insulin sensitivity. The latter was revealed by a higher IR index and increased insulin in the plasma, whereas blood glucose levels remained unchanged. Corticosterone treatment induced longer immobility times in the FST, reflecting depressive-like behaviour. No effects were observed upon anxiety as measured in the EZM. The effect of the higher body weight of the CORT treated animals at time of testing did not influence behaviour in the EZM or FST, as no differences were found in general locomotor activity. Long-term corticosterone treatment via the drinking water reduces insulin sensitivity and induces depressive-like behaviour in the C57BL/6 mouse. This mouse model could thus be used to further explore the underlying mechanisms of chronic stress-induced T2DM and its association with increased prevalence of major depressive disorder on the short-term and other behavioural adaptations on the longer term.

## Introduction

Chronic stress or long-term glucocorticoid administration, as well as (genetic) impairments in HPA-axis or corticoid receptor function are proposed to participate in the aetiology and progression of many health problems, including psychiatric disorders such as major depression and anxiety. Numerous studies have documented hypercortisolaemia, HPA-axis hyperactivity and reduced stress responsivity in patients with depression [1]. Moreover, both environmental and genetic risk factors for depression appear to correlate with increased HPA-axis activity in adulthood [2] and a direct relationship has been observed between peripheral cortisol levels and the severity of depressive symptoms [3].

One of the major metabolic effects of chronic hypercortisolaemia and a key feature in the development of the metabolic syndrome is insulin resistance (IR), a condition characterized by persistent high levels of peripheral glucose (hyperglycaemia) and insulin (hyperinsulinaemia) due to a reduced ability of insulin to lower blood glucose levels by uptake into muscle tissue (insulin insensitivity) [4]. Chronic peripheral hyperinsulinaemia, the so-called pre-diabetic state, can eventually develop into Type 2 diabetes mellitus (T2DM). In addition, glucocorticoids can induce IR in the hippocampus, which explains why the neurological consequences observed in experimental models of T2DM are similar to those observed following chronic stress. Moreover, longitudinal and cross-sectional associations exist between mood disorders and T2DM [5] and reveal that the prevalence of T2DM is increased by two-fold in persons with mood disorders and vice-versa [6]. Glucocorticoids may thus be common mechanistic mediators in the pathophysiological consequences of both T2DM and stress-related disorders [7]. In addition, stress-induced insulin dysregulation and affective disturbances are risk factors for the development of Alzheimer’s disease (AD) [8]–[14]. Recent evidence suggests that cognitive impairment in diet-induced insulin resistant rats may result from glucocorticoid-mediated deficits in neurogenesis and synaptic plasticity [15]. Additionally, glucocorticoids have been shown to aggravate tangle-formation in the hippocampus [16] and affect aβ-processing [17] in rats. Moreover, insulin resistance and the subsequent impairment of insulin signalling in the brain affects the expression and metabolism of Aβ and tau protein [8], [18]–[20]. However, the exact link between chronic stress, major depression and AD has not been established yet and the underlying mechanisms remain unclear [21]–[23].

In mice, long-term daily injections with corticosterone induces depressive-like behaviour and has thus been proposed as a reliable mouse model for studying the implication of stress and glucocorticoids in depression [24]. Insulin resistance has been studied in rats by long-term treatment with low doses of the synthetic glucocorticoid dexamethasone [25] and in mice, similar changes as those observed in individuals suffering from the metabolic syndrome were observed after a long-term, high dose of corticosterone treatment via the drinking water [26]. However, the combined effect of chronic elevated levels of glucocorticoids upon glucose-insulin regulation and depressive-like behavioural changes in rodents remains unclear. A mouse model that mimics chronic stress-induced glucose-insulin dysregulation not only allows for the exploration of acute behavioural changes, but additionally provides the opportunity to link the short-term system adaptations to potential long-term physiological and behavioural consequences of peripheral and central metabolic changes. The present study, therefore, aimed to establish a mouse model of chronic stress-induced insulin resistance and study its effects upon affective behaviour. The proposed model seems suitable, also for potential future application in specific transgenic mouse models.

## Materials and Methods

### Animals

Virgin female C57BL/6NCrl mice (Charles River, L’Arbresle, France) were crossed with male APPswe/PS1ΔE9 mice (Jackson Laboratory, strain #005864, Bar Harbor, ME, USA) at age 8–12 weeks as described by Sierksma *et al*. [27]. A total of 52 male wild type offspring was used of which 12 were handled at age 4-months-old and included for an initial study into the potential of a chronic stress model to induce glucose/insulin dysregulation. The pilot study aimed to establish the optimal administration route for CORT treatment, i.e., orally via drinking water compared to daily subcutaneous (s.c.) injection. In addition, treatment outcome was used to establish a pragmatic measurement of IR that could easily distinguish between normal and insulin resistant animals. Forty mice, age 6-month-old, were used for the main study of which experimental procedures were based upon the treatment results of the pilot study.

Experimental protocols were reviewed and approved by the local ethical Committee on Animal Experimentation of the Maastricht University (DEC2010-190, DEC 2010-010) and met Dutch governmental guidelines. Throughout all experiments the principles of laboratory animal care were carefully followed in accordance with the official codes of practice guidelines (Experiments on Animals Act, The Netherlands, revised, 1996) and all efforts were made to minimize the number of mice used and their suffering.

Mice were individually housed on sawdust bedding in standard Macrolon cages and placed in an individually ventilated cage (IVC) unit, regulating temperature (±20°C) and humidity (40–60%). They were further maintained on a reversed 12∶12-hr light-dark schedule (lights on from 18∶00 h−06∶00 h) and continuous background noise was provided by a softly playing radio. Food (Ssniff, Germany) and tap water were available *ad libitum* throughout the experiments, unless otherwise stated. Animal cages were supplied with paper rolls and tissues to enrich environmental conditions. Clean cages were provided on a weekly basis and mice were weighed weekly and prior to oral glucose administration (*pilot study*). Drinking water consumption was measured by weighing the bottles before and after refreshing them.

To facilitate environmental adaptation, mice were not treated or handled until several weeks after single housing on a reversed day-night cycle. During this initial adaptation period, mice were additionally habituated to general handling procedures, including weighing. For the pilot study, mice were also familiarized to oral (saline) administrations by gavage for subsequent oral glucose administration procedures. At all times, blood samples were taken by means of saphenous vein puncture, as this method has been proven to be appropriate for repeated sampling in the same animal in a quick and painless manner and without the requirement of anesthesia [28]. Mice had both hind paws shaved over the lateral saphenous vein, using a small and almost soundless hair cutting system (Kyone, model GT 100, The Netherlands). For shaving and blood sampling, the animals were placed in a thick cotton cloth, in such a way that the hind paws were freely accessible for the particular procedures. Habituation to the specific restraint procedures and hind paw shaving was included in the initial habituation period as mentioned above.

**Figure 1:**
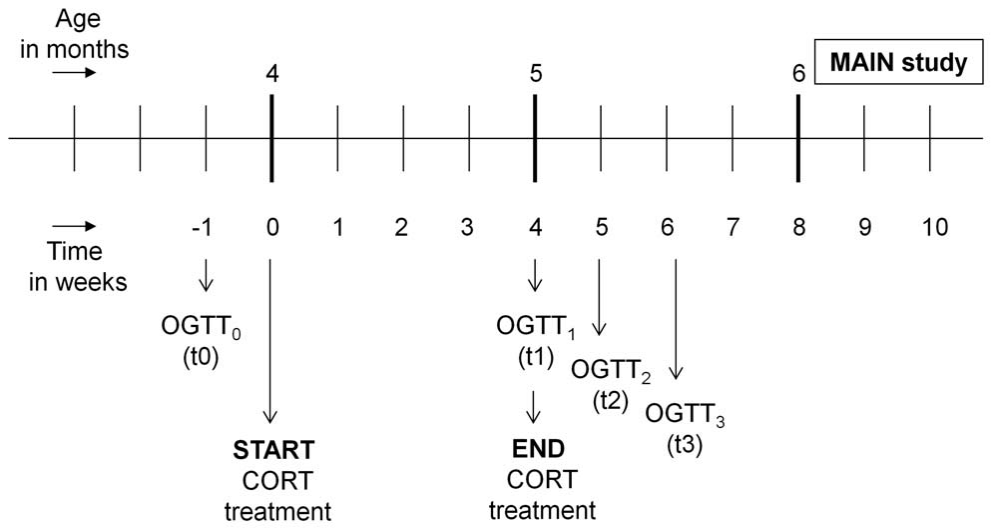
Experimental overview of the pilot study. CORT: corticosterone; OGTT: oral glucose tolerance test.

**Figure 2:**
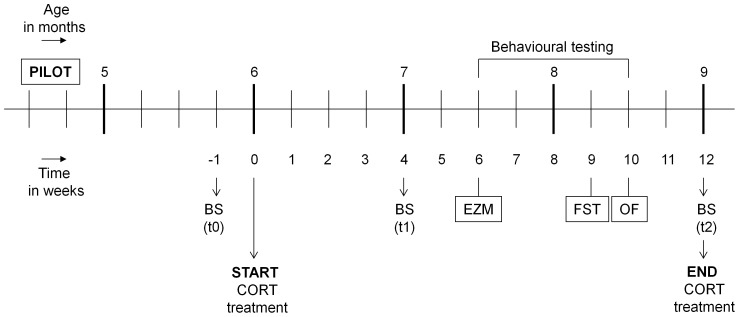
Schematic diagram of the different stages of the main study. BS: blood sample; CORT: corticosterone; EZM: elevated zero maze; FST: forced swimming test; OF: open field test.

Behavioural testing for the main study did not start until 6 weeks into CORT treatment, at approximately 7.5 months of age. This was done deliberately, to make potential future comparisons with the behavioural and cognitive effects in the APPswe/PS1ΔE9 possible. Testing was always done in the (active) dark phase of the rodent day-night cycle. Mice were initially exposed to the elevated zero maze (EZM), followed by the forced swim test (FST) and open field test (OF), with a three-week and one-week interval, respectively (see also Figure 1 and 2 for experimental overviews).

### Corticosterone treatment

Pure (≥92%) corticosterone (CORT) was used, obtained from SIGMA-ALDRICH (Zwijndrecht, The Netherlands). For the pilot study, mice were administered with either CORT (20 mg/kg; n = 3) or vehicle (50% propylene glycol; n = 3) via daily subcutaneous (s.c.) injection or received CORT (100 µg/ml; n = 3) or vehicle (1% ethanol; n = 3) in the drinking water for a total amount of 4 weeks. As CORT is hydrophobic, it was dissolved in ethanol and added to the drinking water to make a 1% ethanol solution. As ethanol is not an appropriate solvent for injectables, CORT was dissolved in propylene glycol and further diluted with saline for the s.c. injections. The vehicle injection group was administered with a 50% propylene glycol solution in saline (5 ml/kg). Injections were alternated daily between left or right flank or over the shoulders (into the loose skin over the neck) to avoid discomfort produced by the procedure or injection-site infection/local skin reaction. Potential interfering ethanol effects in the vehicle drinking water group compared to the other 3 experimental groups were avoided by providing all mice with 1% ethanol in the drinking water.

In the main study, CORT (100 µg/ml; n = 20) or vehicle (1% ethanol; n = 20) was administered via the drinking water for a total time of 12 weeks. Water bottles containing CORT were covered with tinfoil to avoid light-induced degradation of the compound and water quantity was monitored several times throughout the week. When calculating weekly water intake, any water spillage when removing bottles from cages was systematically taken into account by applying a correction factor. This factor was calculated by taken the mean spillage of water after taking the bottle out of the cage for 7 times in a row.

### Insulin sensitivity assessment

#### Glucose tolerance test

The effects of CORT treatment (daily s.c. injection versus drinking water) on insulin sensitivity were evaluated by means of oral glucose tolerance tests (OGTT). An OGGT consists of assessing the efficacy of glucose clearing by insulin in the blood by measuring blood glucose and/or plasma insulin levels at different time points after an oral (body weight-matched) dose of glucose. Mice were fasted for 6 h after which a glucose solution (D-(+)-Glucose, SIGMA-ALDRICH, Zwijndrecht, The Netherlands) of 250 mg/ml was administered orally by gavage (2 g/kg) as derived from Adrikopoulos et al. [29]. Blood samples were taken just prior to glucose administration (baseline) and 15, 30, 60 and 120 minutes after glucose administration, as adapted from Ferrannini and Mari [30] and described by Gounarides et al. [31]. The first drop of blood was removed, the second drop was placed on a test strip (GLUCOCARD X-SENSOR Test Strips) for immediate glucose measurement with a hand-held glucose meter (GLUCOCARD X-meter, A. MENARINI Diagnostics, Benelux, N.V., Valkenswaard, The Netherlands). A subsequent blood sample (±20 µl) was collected in a heparinized microcentrifugation tube for plasma insulin measurement. In order to separate the plasma, blood samples were kept on ice, then centrifuged at 2000 rpm at 4°C for 20 min and finally stored at −80°C until further analysis. Plasma insulin levels were measured using an ultra sensitive mouse insulin ELISA Kit (Crystal Chem, inc., Downers Grove, USA). OGTT was performed prior to the start of the CORT treatment (OGTT0; baseline) and immediately after 4 weeks CORT treatment (OGTT1). To measure the persistence of treatment effects upon glucose tolerance, OGTTs were repeated 1 (OGTT2) and 2 weeks (OGTT3) after treatment cessation.

#### Homeostasis model assessment of insulin resistance index

The homeostasis model assessment of insulin resistance index (HOMA-IR), as described by Matthews *et al*. [32], is the most easily obtained measurement of insulin resistance (IR) and was used as a surrogate measure of *in vivo* insulin sensitivity. Based upon fasting blood glucose and plasma insulin levels (baseline, i.e. the blood sample prior to glucose administration), the following formula is used: HOMA-IR = fasting [insulin(mIU/l)]*fasting [glucose(mmol/l)]/22.5. Lee et al. [33] found that the HOMA-IR index correctly differentiated between insulin sensitivity and IR. In the drinking water condition, in addition to the OGTT measurements for insulin sensitivity assessment, HOMA-IR indices were calculated and compared for CORT effects directly after treatment (OGTT1; t1) and 1 (OGTT2; t2) and 2 weeks (OGTT3; t3) after treatment cessation. Blood samples for fasting blood glucose and plasma insulin samples were the same samples as those obtained from OGTT1, 2 and 3 at time 0 (baseline; prior to oral glucose administration).

Based upon the results of the effects of CORT treatment via daily s.c. injection versus drinking water upon body weight, glucose tolerance and insulin sensitivity, it was decided to rely on the HOMA-IR index for insulin sensitivity measurements in the main study. Baseline blood glucose and plasma insulin levels were measured at 3 different time-points throughout the main study, namely prior to the start of CORT treatment (baseline; t0) and 4 (t1) and 12 weeks (t2) into CORT treatment. Blood glucose and plasma insulin levels were used to calculate the HOMA-IR index as described earlier (see Figure 2 for an overview of the experimental procedures during the main study).

### Behavioural testing

#### Elevated zero-maze test

The elevated zero-maze test (EZM) is a modification of the elevated plus-maze and models anxiety-like behaviour in rodents [34]. The maze consisted of a circular platform (50 cm in diameter), elevated 20 cm above floor level, with two opposite enclosed parts (50 cm high side walls) and two open parts equally divided along the circular runway (5 cm). This modified design allows the animal uninterrupted exploration and avoids undefined time spent in the central square as is the case in the traditional elevated plus-maze design [34]. Falls from the open parts were prevented by a 5 mm high edge. Both the side walls and the maze itself were made of black plastic, transparent for infrared light, and connected via an infrared video camera to a video tracking system (Ethovision Pro, Noldus Wageningen, The Netherlands). For testing, mice were placed in the middle of one of the open parts, facing a closed part, and allowed to explore the maze for a total time of 5 min. In between trials, the maze was thoroughly cleaned with water and 70% alcohol. Total time spent in closed and open parts and total distance travelled was video tracked and the percentage time spent in the open arms was calculated and corrected for the first latency to enter a closed arm.

#### Forced swimming test

A modified forced swimming test (FST) consisted of exposing the animals to an inescapable water stress, using the total time spent in an immobile position as a measure of their level of behavioural despair [35], [36]. For the swim sessions, transparent Plexiglas cylinders (50 cm in height×19 cm in diameter), those normally used for rats, were filled with warm water (32±2°C) up to 20 cm. Water height was specifically chosen to prevent the mice from being able to touch the cylinder bottom with their hind paws or tail [37], [38]. Four mice were tested in parallel and videotaped from above. Mice were not able to see one another due to grey separation panels placed between the four cylinders. The 6 min swim sessions were initiated by transferring the mice from their home cage to one of the cylinders. After each session, mice were removed from the water and transferred to a cage filled with paper towels for an initial 2 min recovery. Subsequently, they were dried with paper towels and returned to their home cages. In between trials, cylinders were cleaned with 70% alcohol and refilled with clean water.

The cylinders were positioned on top of a light box to create optimal illumination conditions for video tracking. A camera was placed 75 cm above the light box and connected to a video tracking system (Ethovision Pro, Noldus Wageningen, The Netherlands), which allowed automated recording of the total distance moved. Total immobility time for each mouse was scored manually by an independent researcher blind for experimental conditions.

#### Open field test

The open field (OF) was originally designed to simultaneously measure locomotion, exploration and anxiety by exposing mice to a novel and open space [39]. For the present study, only general locomotor activity was scored to be able to control for potential interfering effects due to differences in body weight. Due to the relatively small dimensions of the separate arenas, the test was not found appropriate for assessing additional anxiety-like behaviour in a valid manner [40]. Therefore, total time spent in corners, periphery or centre of the arena was not taken into account. However, defecation was used as indicator of emotionality/anxiety and was measured by the number of faecal boli produced during the test session.

The equipment was made of acrylic and consisted of a grey square arena (50×50×25 cm), subdivided by grey walls (height: 25 cm) into 4 separate arenas of 25×25×25 cm. Four mice were tested in parallel by placing them in the centre of the arenas, allowing them to move around freely for a total time of 20 min. After each 20 min session, faecal boli were counted per arena/mouse and arenas were subsequently thoroughly cleaned with 70% alcohol. Sessions were run under dimmed lightning conditions and videotaped from above by a camera connected to a video tracking system (Ethovision Pro, Noldus Wageningen, The Netherlands) which allowed automated tracking of the total distance moved for each mouse separately.

### Statistics

In the pilot study, changes in body weight and glucose tolerance over time were analyzed by means of a three-way analysis of variance (ANOVA) with Treatment and Administration Route as between-subject factors and Time as within-subject variable. In all other cases, changes over time were analyzed by means of two-way ANOVA with Treatment as between-subject factor and Time as within-subject variable. Separate one-way ANOVAs or independent Student’s *t*-tests were performed to analyze differences between treatment conditions per time-point and the effects upon behaviour. Where appropriate, a post hoc Bonferroni test was used to further characterize the treatment effects.

In all cases the acceptable level of significance was set at *P*<0.05. All data are represented as mean ± standard error of the mean (S.E.M.). Analyses were performed using the Statistical Package for Social Science (SPSS) for Windows (IBM SPSS Statistics version 20). GraphPad Prism, version 4.02 for Windows was used for preparing the graphs.

## Results

### Pilot study

#### Body weight

Significantly increased body weights were found over the course of 6 weeks [*F*(5, 40) = 18.19; *P*<0.001]. However, changes over time depended on treatment [*F*(5, 40) = 7.15; *P*<0.001] and route of administration [*F*(5, 40) = 5.35; *P*<0.01]. Moreover, a significant time*treatment*route interaction was found [*F*(5, 40) = 2.97; *P*<0.05]. An overall treatment effect [*F*(1, 8) = 5.85; *P*<0.05] and effect of administration route [*F*(1, 8) = 15.37; *P*<0.01] was revealed, however, no significant treatment*route interaction was observed [*F*(1, 8) = 3.21; n.s.].

**Figure 3:**
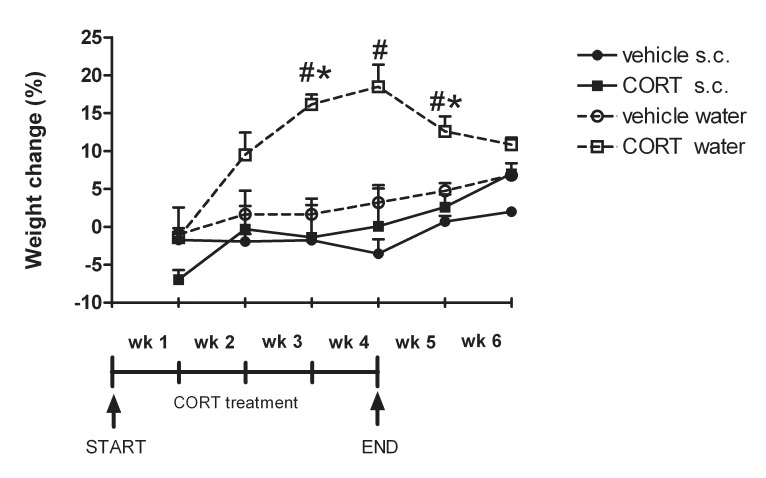
Effects of CORT treatment orally via the drinking water or by daily s.c. injection upon body weight (mean % change compared to body weight prior to start of treatment+S.E.M.) during the 4 week treatment period and 1 and 2 weeks after treatment cessation (week 5 and 6). The figure shows significant increased body weights in the group receiving CORT via the drinking water compared to the vehicle drinking water group (^#^) or CORT injection group (*). ^#^/*P<0.05.

Separate one-way ANOVAs for treatment effects per week revealed no differences in body weight change at 1 [*F*(3, 11) = 1.89; n.s.] and 2 weeks [*F*(3, 11) = 2.80; n.s.] into CORT treatment. Significantly increased body weights were found after 3 weeks CORT treatment body weights were significantly increased [*F*(3, 11) = 9.73; *P*<0.01] with higher body weights in the CORT drinking water condition compared to the vehicle drinking water group and the group receiving CORT via injection as revealed by post hoc analyses. The same was true at the end (after 4 weeks) of CORT treatment [*F*(3, 11) = 8.34; *P*<0.01] with post hoc analyses showing significantly higher body weights in mice receiving CORT via the drinking water compared to mice receiving daily CORT injections. Only a trend was found toward increased body weights in the mice receiving CORT through drinking water compared to their vehicle counterparts (*P* = 0.07). One week after treatment cessation (week 5) body weights of mice receiving CORT via drinking water were still significantly higher compared to all other groups [*F*(3, 11) = 13.15; *P*<0.01]. Two weeks after treatment cessation body weights were still significantly increased [*F*(3, 11) = 8.34; *P*<0.01], however, post hoc analyses revealed increased body weights only in the CORT drinking water group compared to the vehicle injection group. At this time point, no differences were found between the CORT drinking water and CORT injection condition or between the CORT drinking water and vehicle drinking water group (see Figure 3).

#### Glucose tolerance test

**Figure 4:**
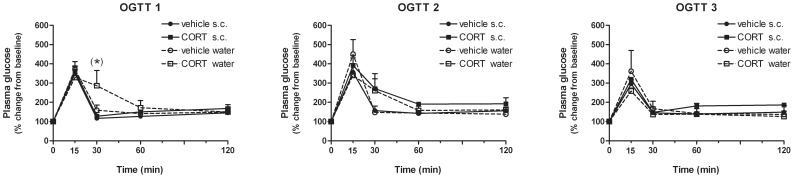
Plasma glucose levels (mean % change from baseline+S.E.M.) after acute glucose challenge in fasted animals receiving corticosterone (CORT) or vehicle either orally via the drinking water or by daily s.c. injection as measured by oral glucose tolerance tests (OGGT). Within each OGTT, plasma glucose levels are measured at baseline (0) and 15, 30, 60 and 120 min after an oral administration of glucose. Treatment effects upon glucose tolerance were measured at 3 time-points throughout the experiment, namely directly after 4 weeks treatment (OGTT1) and 1 and 2 weeks after treatment cessation (OGTT2, OGTT3, respectively). (*) *trend*; *P* = 0.094.

Glucose tolerance prior to the start of CORT treatment (OGGT0) is not depicted in a graph as it involves one baseline measurement of all mice together. Directly after 4 weeks CORT treatment (OGGT1), glucose tolerance differed over time, depending on route of administration [*F*(4, 32) = 4.77; *P*<0.01]. In addition, a tendency towards a time*treatment interaction effect [*F*(4, 32) = 2.40; *P* = 0.070] and time*treatment*route interaction effect [*F*(4, 32) = 2.46; *P* = 0.065] was observed. Subsequent one-way ANOVA for detailed analysis of treatment effects per time-point reveals a trend towards impaired glucose clearance 30 min after oral glucose administration in the CORT via drinking water condition compared to the other treatment groups (*P* = 0.094). During OGTT2, only a significant time*treatment interaction [*F*(2.73) = 2.40; *P* = 0.047] was revealed. Overall treatment effects upon glucose tolerance were not found during any of the OGTTs (see Figure 4).

#### Blood glucose, plasma insulin and HOMA-IR

Although no effects were found of CORT treatment upon fasting blood glucose and plasma insulin levels (independent of administration route), a significant higher HOMA-IR index in the CORT drinking water group compared to the vehicle drinking water group directly after the 4 weeks treatment period (t1) was found [*t*(4) = −3.06; *P*<0.05]. Treatment cessation led to a normalisation as no differences were found in HOMA-IR indices 1 (t2) [*t*(4) = −0.47; n.s.] and 2 weeks after the end of CORT treatment (t3) [*t*(4) = −0.40; n.s.] (Table 1).

**Table 1:** Mean (± S.E.M.) fasting blood glucose (mmol/l), plasma insulin (ng/ml) values and HOMA-IR index directly after 4 weeks treatment via drinking water (t1) and 1 (t2) and 2 weeks (t3) after treatment cessation.

Time	Treatment	Glucose	Insulin	HOMA-IR
t1	vehicle	6.5±0.8	1.0±0.5	6.9±2.8
	CORT	4.9±1.0	8.7±3.9	38.7±10*
t2	vehicle	6.2±0.4	0.7±0.1	4.5±0.2
	CORT	6.3±0.5	0.7±0.1	5.0±0.9
t3	vehicle	8.0±1.4	1.7±0.3	14.5±3.4
	CORT	8.3±0.6	1.8±0.4	16.3±2.7

CORT: corticosterone; HOMA-IR: homeostasis model assessment of insulin resistance index *P<0.05.

### Main study

#### Body weight

**Figure 5:**
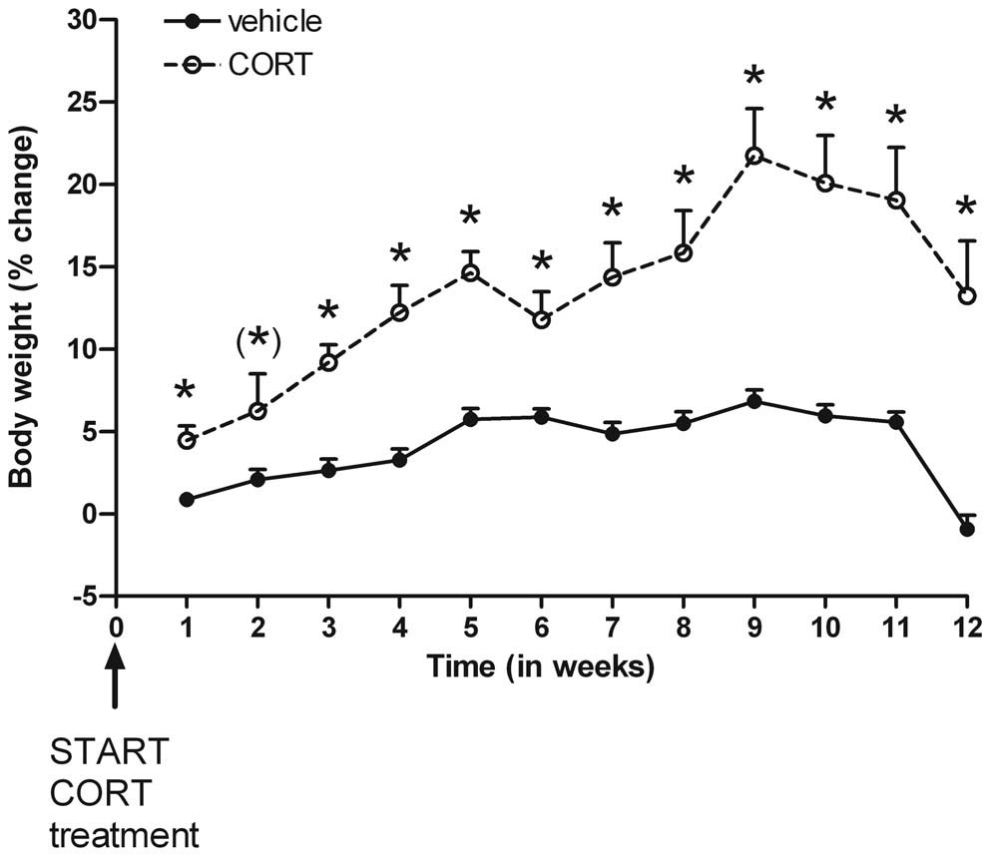
Effects of corticosterone (CORT) administration via the drinking water upon body weight (mean % change compared to body weight prior to start of treatment+S.E.M.) during the 12 week treatment period. Significant increased body weights in the CORT group are denoted with * (P<0.01); (*) *trend.*

Body weight (see Figure 5) significantly increased during the 12 week experimental period [*F*(11, 341) = 21.40; *P*<0.001], but not for both groups (CORT versus vehicle) similarly [*F*(11, 341) = 5.44; *P*<0.001]. An overall treatment effect revealed that mice receiving CORT via the drinking water gained significantly more weight compared to the control vehicle group [*F*(1, 31) = 37.02; *P*<0.001]. Separate *t*-tests per week showed increased body weights in the CORT treated mice at all time points (all *P*<0.01), except for week 2, which resulted in a trend towards increased body weight [*t*(37) = −1.75; *P* = 0.09].

#### Water intake

**Figure 6:**
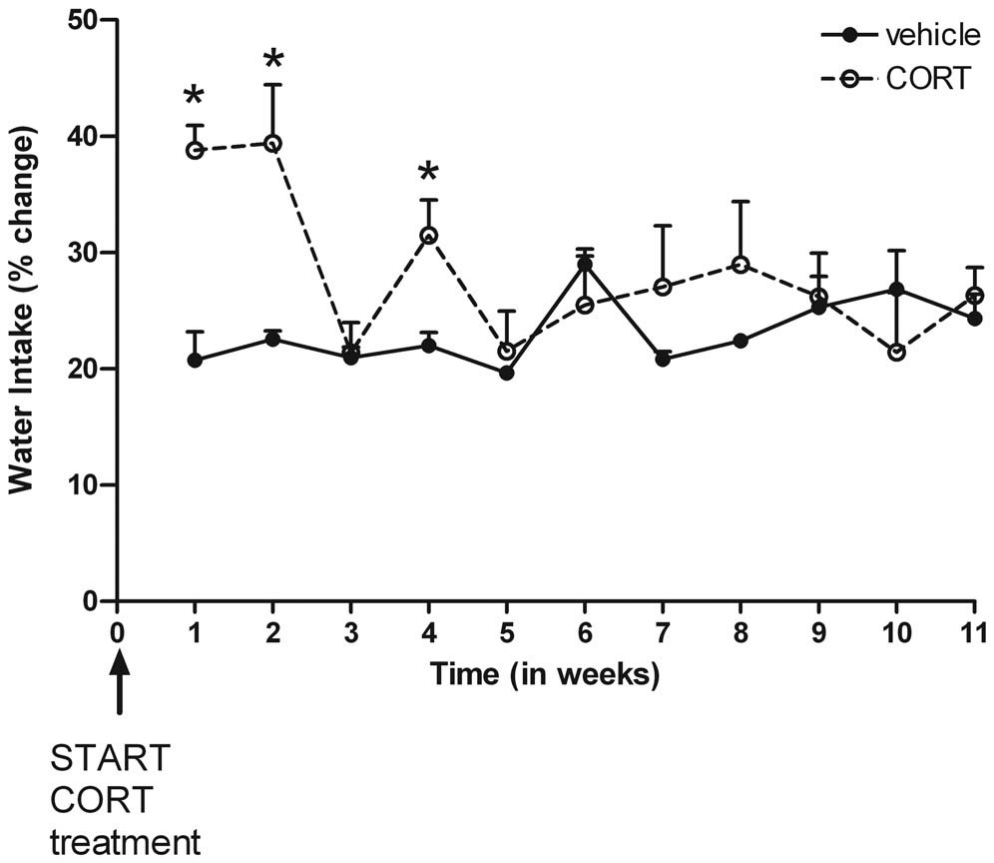
Effects of corticosterone (CORT) administration via the drinking water upon water intake (mean % change+S.E.M.) during the 12 week treatment period. Significant increased amounts of water intake in the CORT group are denoted with *(*P*<0.01).

The amount of water intake changed over time [*F*(10, 280) = 4.06; *P*<0.001] and differently depending on treatment condition [*F*(10, 280) = 6.08; *P*<0.001]. However, no overall treatment effect was found [*F*(1, 28) = 1.15; n.s.]. When analyzing differences between the treatment groups per time point, separate *t*-tests revealed significant higher amounts of water intake only at week 1 [*t*(35) = −5.54; *P*<0.001], 2 [*t*(35) = −3.40; *P*<0.01] and 4 [*t*(35) = −2.92; *P*<0.01] in the CORT treated animals (see Figure 6).

#### Blood glucose, plasma insulin and HOMA-IR

Table 2 shows the effects of CORT treatment upon blood glucose, plasma insulin and HOMA-IR index throughout the experiment. Blood glucose levels significantly increased over time [*F*(2, 62) = 30.84; *P*<0.001], but differently depending on treatment condition [*F*(2, 62) = 12.05; *P*<0.001]. However, an overall treatment effect was not found, but did result in a trend towards increased blood glucose levels in the vehicle treated animals at 12 weeks [*F*(1, 31) = 3.97; *P* = 0.06].

**Table 2:** Mean (± S.E.M.) fasting blood glucose (mmol/l), plasma insulin (ng/ml) values and HOMA-IR index at baseline (t0), after 4 weeks (t1) and after 12 weeks treatment (t2).

Time	Treatment	Glucose	Insulin	HOMA-IR
t0	vehicle	5.0±0.2		
	CORT	5.3±0.2		
t1	vehicle	5.5±0.2	1.1±0.2	6.8±1.1
	CORT	5.4±0.2	6.3±0.8*	37.1±4.5*
t2	vehicle	7.8±0.3	1.0±0.1	8.7±1.2
	CORT	6.1±0.4	3.7±0.9*	24.6±5.3*

CORT: corticosterone; HOMA-IR: homeostasis model assessment of insulin resistance index *P<0.01.

Plasma insulin levels [*F*(1, 19) = 3.06; n.s.] and HOMA-IR index [*F*(1, 19) = 1.44; n.s.] did not change over time, but overall treatment effects were found ([*F*(1, 19) = 39.19; *P*<0.001] and [*F*(1, 19) = 33.17; *P*<0.001], respectively). Separate *t*-tests for treatment effects per time-point revealed significantly increased plasma insulin levels in CORT treated mice compared to the vehicle group after both 4 [*t*(36) = −6.25; *P*<0.001] and 12 weeks [*t*(19) = −3.07; *P*<0.01] of CORT treatment and similar effects were found for the HOMA-IR index after both 4 [*t*(36) = −6.25; *P*<0.001] and 12 weeks [*t*(19) = −3.07; *P*<0.01] of CORT treatment.

#### Behaviour

**Figure 7:**
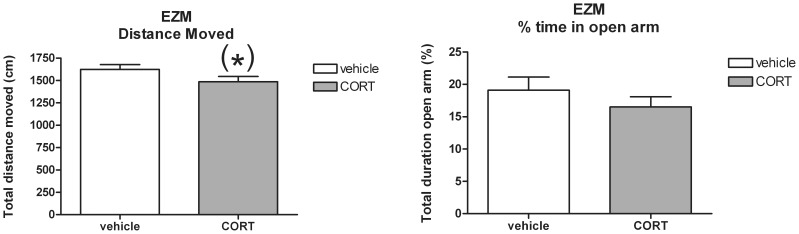
Effect of CORT administration via the drinking water (mean+S.E.M.) upon total distance moved and % time spent in the open arms of the elevated zero maze (EZM). (*) Trend towards difference between CORT and vehicle treatment (*P* = 0.09).

A trend was revealed towards less distance moved by CORT treated mice compared to the vehicle group [*t*(34) = 0.67; *P* = 0.09] in the EZM. However, the percentage of time spent in the open arms was not different between treatment conditions [*t*(34) = 0.60; n.s.] (see Figure 7).

**Figure 8:**
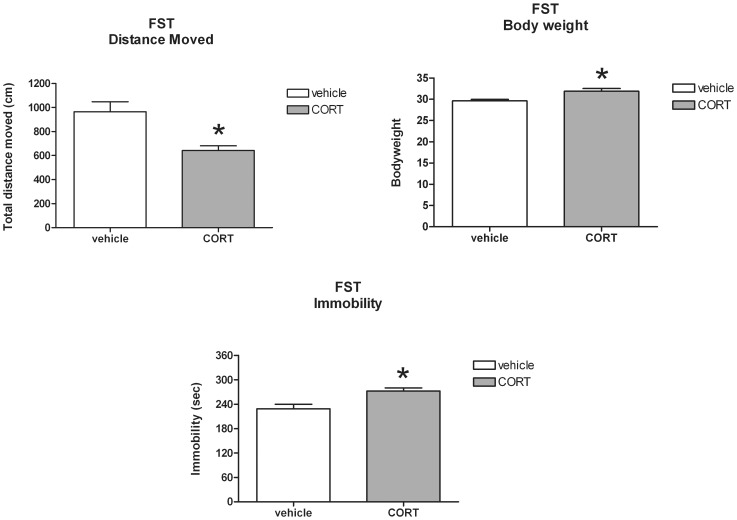
Effect of corticosterone (CORT) administration via the drinking water (mean+S.E.M.) upon total distance moved, body weight at time of testing and total immobility time in the forced swim test (FST). *Significant difference between CORT and vehicle treatment (*P*<0.01).

In the FST, the total distance moved was significantly lower in CORT treated mice [*t*(34) = 3.37; *P*<0.01], whereas body weight at time of testing was significantly higher [*t*(34) = −3.16; *P*<0.01]. In addition, CORT treated mice showed more depressive-like behaviour as was reflected upon by the significantly increased immobility times in this group [*t*(34) = −3.15; *P*<0.01] (see Figure 8).

**Figure 9:**
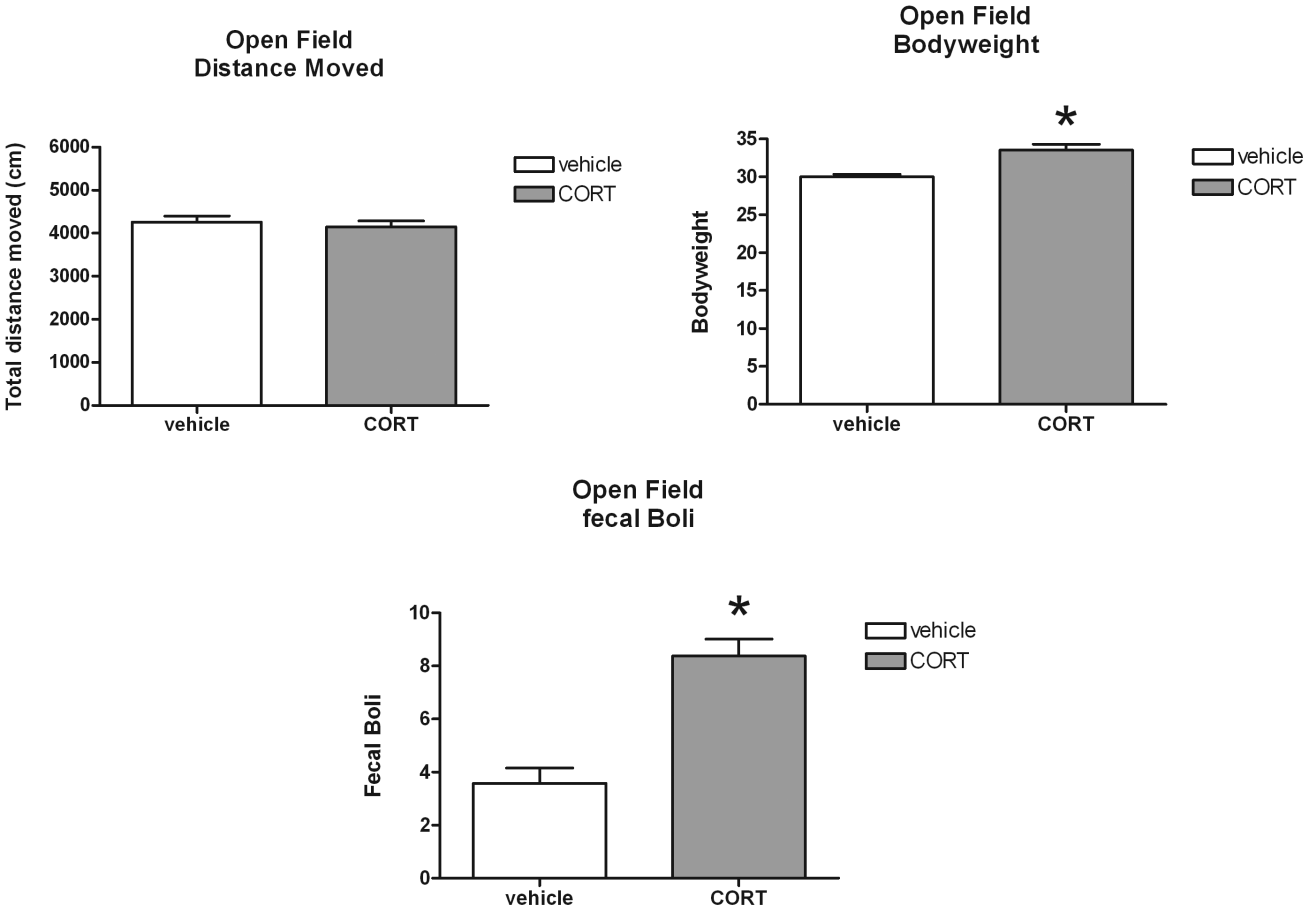
Effect of corticosterone (CORT) administration via the drinking water (mean+S.E.M.) upon total distance moved, body weight at time of testing and fecal boli production in the open field test (OF). *Significant difference between CORT and vehicle treatment (*P*<0.01).

For the OF, no effects of CORT treatment upon total distance moved were found [*t*(33) = 0.55; n.s.], whereas mean body weight of the CORT treated mice, like during the FST, was significantly higher compared to the vehicle group [*t*(34) = −4.57; *P*<0.001]. Moreover, the amount of faecal boli was significantly higher in the CORT treated animals [*t*(34) = −5.63; *P*<0.001] (see Figure 9).

## Discussion

The present study aimed to establish a stress-IR-depression model in the mouse and specifically in the C57BL/6NCrl mouse since it is the background mouse strain used for all behavioural experiments in transgenic and knockout research [41]. To the best of our knowledge, this is the first study to explore the effects of chronic glucocorticoid exposure upon both insulin-glucose regulatory systems and affective behavioural changes in the mouse.

Prior to the main study, 12 mice were used to clarify the difference between continuous CORT exposure via the drinking water or by daily s.c. injection, the reversibility of physiological effects after treatment cessation and the reliability of oral glucose tolerance tests to indicate between-group changes in insulin sensitivity. No effect of 4 weeks CORT treatment via the drinking water or daily injection on glucose tolerance could be revealed. This is not in line with the severely compromised glucose tolerance found by Karatsoreos *et al*. [26] after 4 weeks CORT treatment via the drinking water. With the same dose and mouse strain, Karatsoreos *et al.* [26] observed significantly high plasma glucose levels even 120 min after glucose administration. However, they administered glucose via intraperitoneal (i.p.) injection which avoids the incretin response that potentiates the glucose-mediated insulin response [29] which could explain their higher glucose levels due to a lack of initial insulin secretion. Based upon the observation that a slight tendency was observed towards higher glucose levels 30 min after glucose administration only in the mice that received CORT via the drinking water (present study), together with the results of CORT administration via the drinking water as showed by Karatsoreos *et al*. [26], it was decided to continue further investigation for the drinking water group only. The food-deprivation state prior to glucose administration allowed us to collect fasting blood samples from which, besides blood glucose levels, also plasma insulin levels could be determined. From these parameters the HOMA-IR index was calculated which is a surrogate marker of IR and a useful non-invasive way of determining insulin sensitivity in mice [29]. Despite unchanged glucose and insulin levels, the HOMA-IR index was significantly higher in the CORT treated mice immediately after 4 weeks treatment, indicating reduced insulin sensitivity. These data also showed that treatment cessation reverses the treatment effect, as HOMA-IR index was back to normal levels 1 and 2 weeks after the end of treatment. Although it should be taken into account that the above mentioned pilot data is derived from only 3 animals per experimental group, the data served to provide information concerning the experimental protocol of the main study. CORT treatment was administered via drinking water and continuously throughout the entire experiment for valid behavioural assessment. Moreover, HOMA-IR index could be used as non-invasive measure to assess changes in insulin sensitivity instead of the relatively invasive OGTT procedure.

Daily s.c. or i.p. injections of glucocorticoids solutions might be more precise and standardized with respect of the amount of drug delivered at a specific time point thereby avoiding fluctuations of circulating drug concentrations. However, the acute stress effect of the injections can easily and seriously confound the long-term effects of continuous high levels of glucocorticoids [42]. Moreover, administering CORT via the drinking water is not only a technically low demanding and non-invasive method, it additionally allows for the maintenance of the naturally occurring diurnal rhythm of the hormone secretion [26]. Despite some inexplicable fluctuations in water intake at the very beginning of the treatment period, no difference in amount of intake was found between the treatment groups overall. All animals thus received the same amount of water and CORT over a one week period, i.e. until the weighing and refreshing of the drinking bottles. Throughout the entire 12 week treatment period, CORT treated mice significantly increased their body weight, which is in line with the observation that chronic stress in humans has been linked to obesity [43]. Unfortunately, the amount of food consumed was not taken into account for the present study. However, Karatsoreos *at al*., [26] found similar increases in mouse body weight after 4 week continuous CORT treatment via the drinking water which was clearly attributable to CORT-induced hyperphagia with a significantly increased proportion of food consumed by body weight. They additionally report increased levels of plasma leptin which indicates a degree of resistance to the action of leptin which normally has an important role in the regulation of food intake.

In addition, the present study shows that CORT treatment significantly increased plasma insulin concentrations, whereas blood glucose levels remained unchanged. Insulin works as a key signal of blood glucose levels and mean plasma insulin levels can therefore be used to indicate the relative degree of insulin sensitivity with a higher plasma insulin concentration reflecting more insulin resistance [30]. Thus, the higher levels of insulin as observed in the present study, together with the unchanged levels of glucose in the blood suggest the development of resistance to the action of insulin as is also seen in T2DM. This was confirmed by the significantly higher HOMA-IR index in the CORT treated mice. However, although peripheral insulin resistance is one of the hallmarks of T2DM, development of diabetes additionally requires the onset of β-cell dysfunction [44]. Decreased insulin sensitivity together with increased body weight are features that nicely resemble some of the key physiological effects of hypercortisolaemia as observed in people suffering from Cushing’s syndrome (excess of cortisol secretion) or the metabolic syndrome. Besides glucose intolerance and insulin resistance, the metabolic syndrome is further characterized by (abdominal) obesity, dyslipidemia and hypertension and increases the risk to develop T2DM or cardiovascular disease [45]. The results of the present study thus confirm the observation of Karatsoreos *et al.* [26], that treating mice with a relatively high dose of CORT (100 µg/ml) via the drinking water continuously for at least 4 weeks serves as a valid model of key features of the metabolic syndrome in the mouse, which precedes T2DM.

Chronic stress-induced HPA-axis dysfunction is well known to increase the risk to developing major depression [1] and the prevalence of T2DM appears to be increased by two-fold in persons with mood disorders and vice-versa [6]. The present study, therefore, assessed the effect of long-term CORT treatment upon both glucose-insulin dysregulation and affective behavioural changes. Whereas prolonged CORT treatment did not induce anxiety, significant increased immobility times were observed in the FST, reflecting depressive-like behaviour [46]. Similar increases in FST immobility times after prolonged glucocorticoid exposure in C57BL/6 mice have also been found by others, both after sub-chronic treatment (3 and 5 weeks) with daily s.c. injections [24] and after continuous exposure via the drinking water for either 2 weeks [47] or 4 weeks [48]. In line with this, decreased grooming behaviour [48] and a state of anhedonia, as measured by sucrose preference, has been reported [47], [48]. Moreover, significant increased defecation (fecal boli) was observed during a 20 min open field session, indicative of disturbed emotionality rather than signs of fearfulness or anxiety [49]. Although at time of behavioural assessment, CORT treated mice had significantly higher body weights, behavioural effects cannot be attributed to differences in body weight as no differences in general locomotor activity (total distance moved) were observed in the OF.

### General conclusion

Long-term continuous treatment with exogenous CORT via the drinking water in the mouse seems a valid and reversible model of decreased insulin sensitivity mimicking both the physiological and the behavioural effects of hypercortisolaemia-induced IR which can ultimately develop in T2DM. The model therefore serves to further explore the causal direction and underlying mechanism of the strong association between IR and major depression, which up until today remains unresolved. As the model was established in C57BL/6NCrl mice, which is the genetic background mouse of many genetically manipulated mouse strains, including the APP/PS1 Alzheimer mouse, it additionally allows further investigation into the long-term effects of stress-induced IR and depression as risk factors for developing Alzheimer’s disease later in life.
